# Supplementary material for: Response of Horticultural Soil Microbiota to Different Fertilization Practices
Source: Plants (Basel). 2020 Nov 6;9(11):1501. doi: 10.3390/plants9111501 (PMC7694448; doi:10.3390/plants9111501)
Supplement: Supplementary file 1 [file plants-09-01501-s001.zip › plants-964489-SI.pptx]

## Slide 1
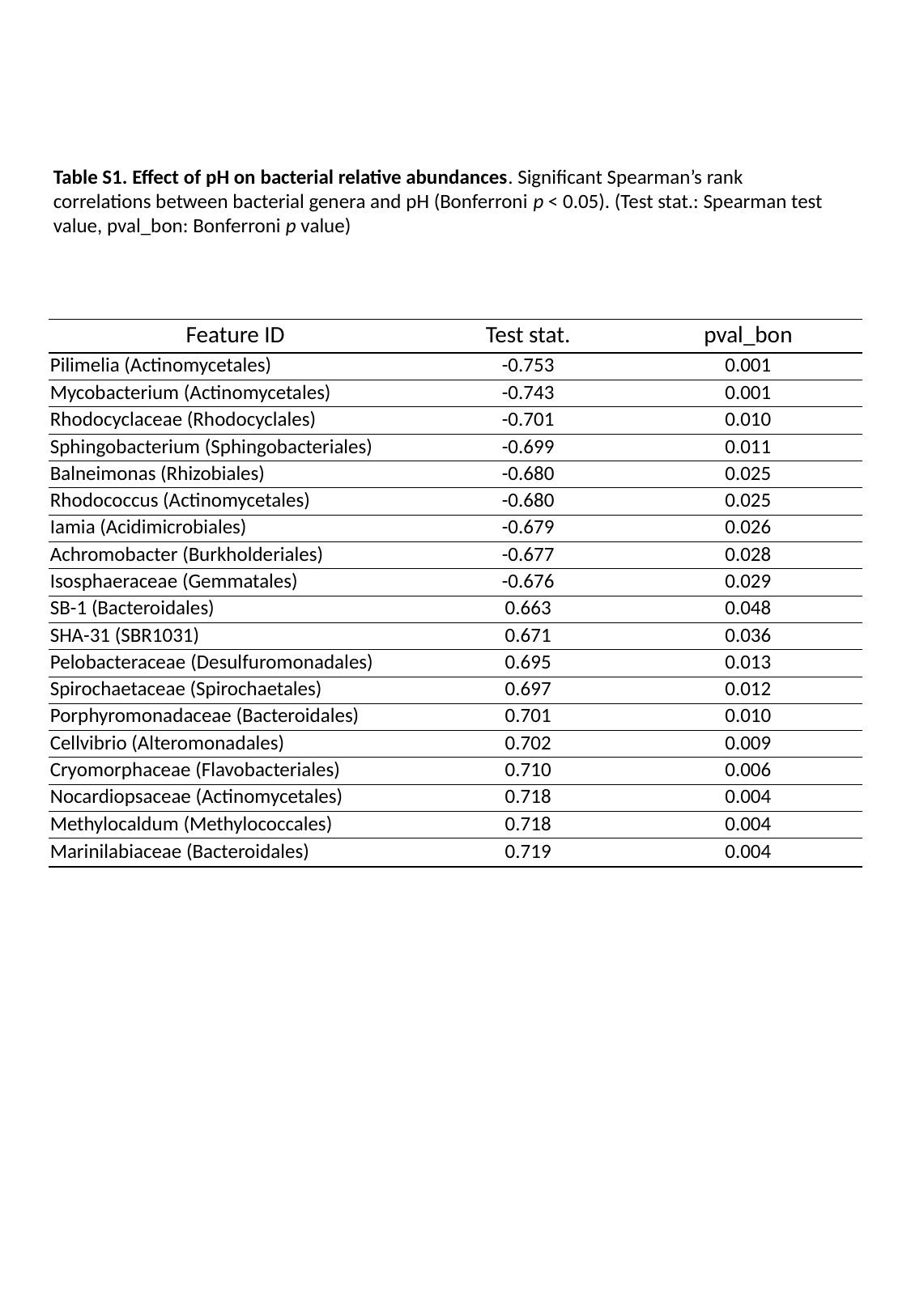

Table S1. Effect of pH on bacterial relative abundances. Significant Spearman’s rank correlations between bacterial genera and pH (Bonferroni p < 0.05). (Test stat.: Spearman test value, pval_bon: Bonferroni p value)
| Feature ID | Test stat. | pval\_bon |
| --- | --- | --- |
| Pilimelia (Actinomycetales) | -0.753 | 0.001 |
| Mycobacterium (Actinomycetales) | -0.743 | 0.001 |
| Rhodocyclaceae (Rhodocyclales) | -0.701 | 0.010 |
| Sphingobacterium (Sphingobacteriales) | -0.699 | 0.011 |
| Balneimonas (Rhizobiales) | -0.680 | 0.025 |
| Rhodococcus (Actinomycetales) | -0.680 | 0.025 |
| Iamia (Acidimicrobiales) | -0.679 | 0.026 |
| Achromobacter (Burkholderiales) | -0.677 | 0.028 |
| Isosphaeraceae (Gemmatales) | -0.676 | 0.029 |
| SB-1 (Bacteroidales) | 0.663 | 0.048 |
| SHA-31 (SBR1031) | 0.671 | 0.036 |
| Pelobacteraceae (Desulfuromonadales) | 0.695 | 0.013 |
| Spirochaetaceae (Spirochaetales) | 0.697 | 0.012 |
| Porphyromonadaceae (Bacteroidales) | 0.701 | 0.010 |
| Cellvibrio (Alteromonadales) | 0.702 | 0.009 |
| Cryomorphaceae (Flavobacteriales) | 0.710 | 0.006 |
| Nocardiopsaceae (Actinomycetales) | 0.718 | 0.004 |
| Methylocaldum (Methylococcales) | 0.718 | 0.004 |
| Marinilabiaceae (Bacteroidales) | 0.719 | 0.004 |

## Slide 2
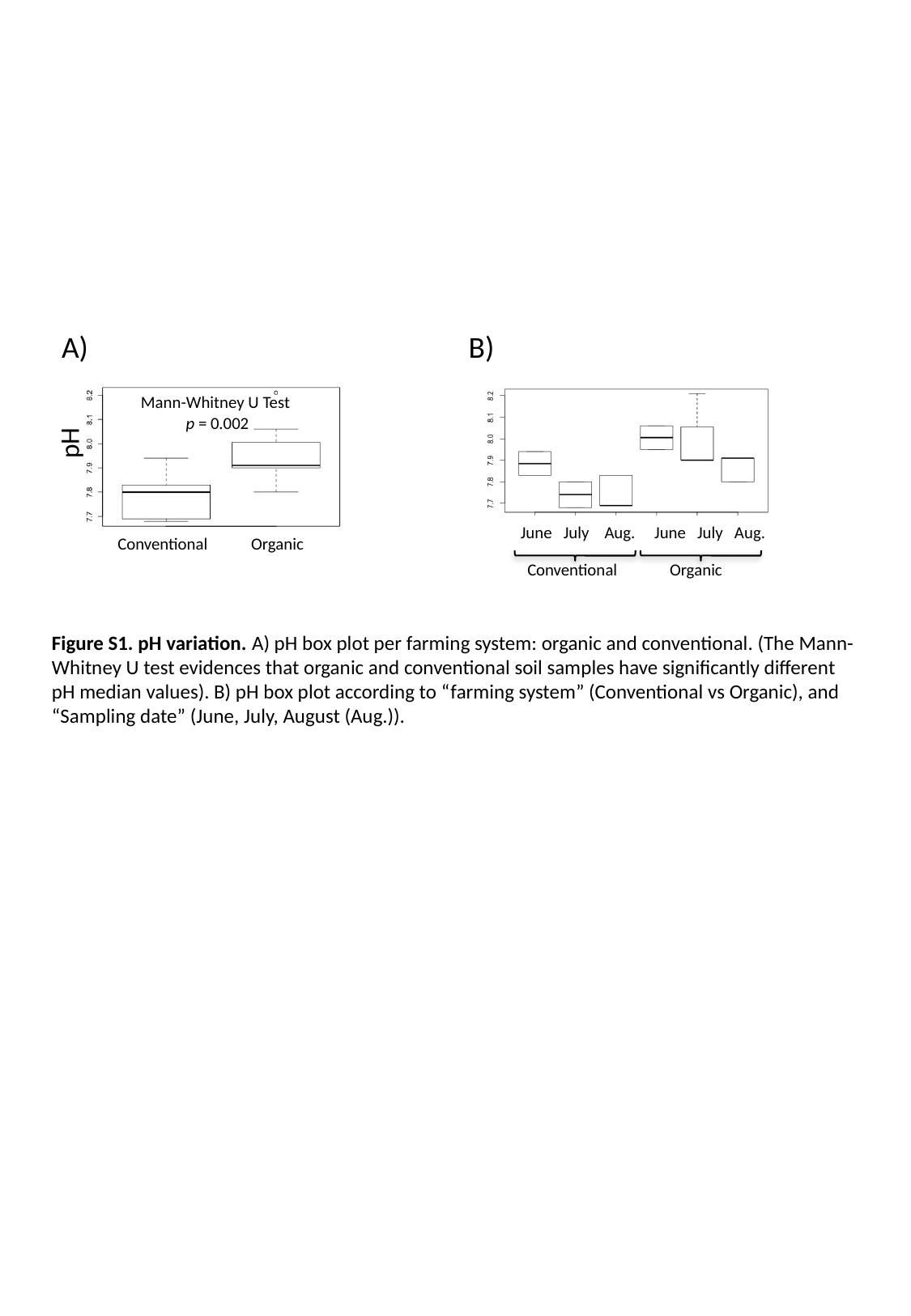

A)
B)
Mann-Whitney U Test
 p = 0.002
pH
Organic
Conventional
 June July Aug. June July Aug.
Conventional
Organic
Figure S1. pH variation. A) pH box plot per farming system: organic and conventional. (The Mann-Whitney U test evidences that organic and conventional soil samples have significantly different pH median values). B) pH box plot according to “farming system” (Conventional vs Organic), and “Sampling date” (June, July, August (Aug.)).

## Slide 3
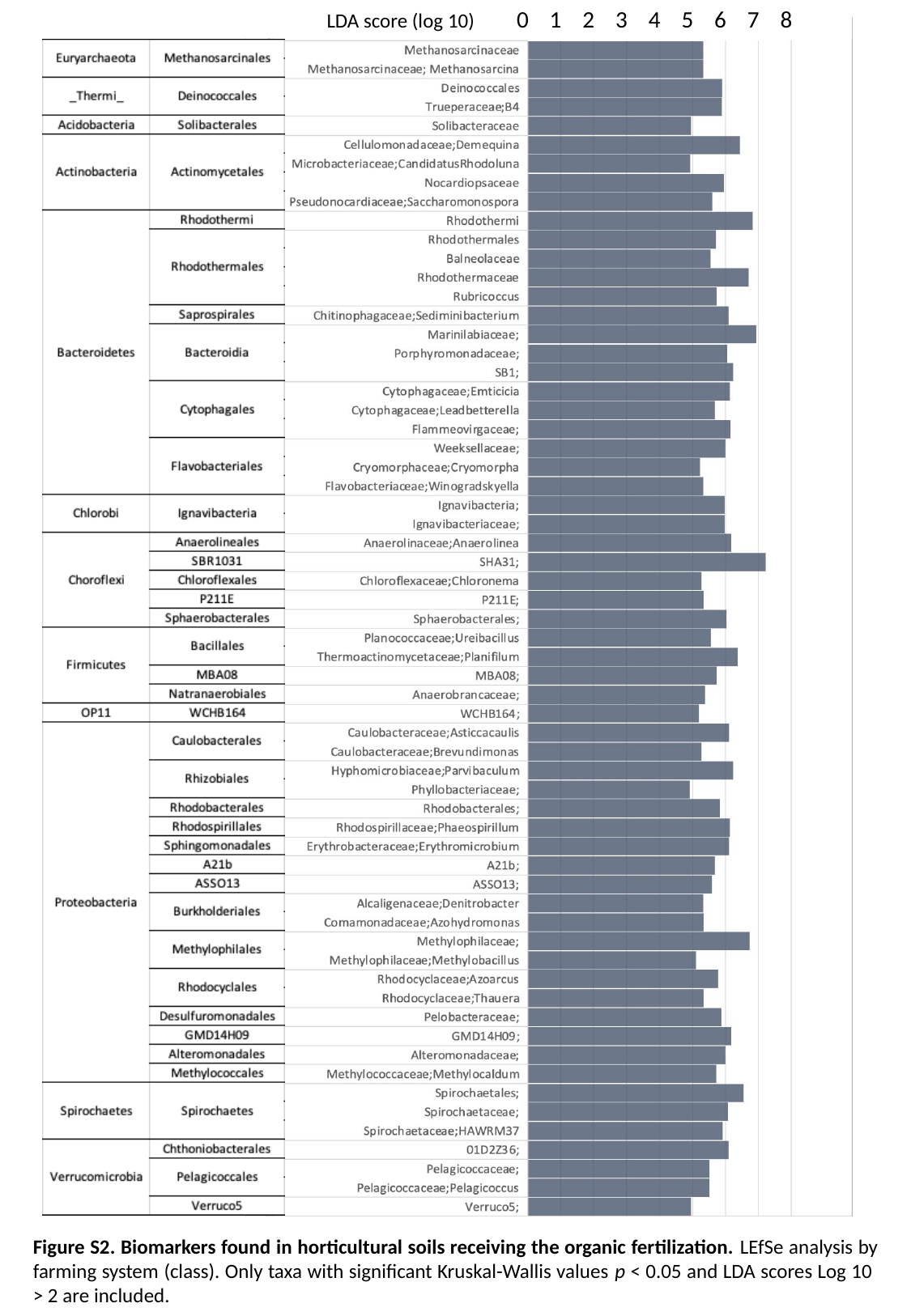

LDA score (log 10) 0 1 2 3 4 5 6 7 8
Figure S2. Biomarkers found in horticultural soils receiving the organic fertilization. LEfSe analysis by farming system (class). Only taxa with significant Kruskal-Wallis values p < 0.05 and LDA scores Log 10 > 2 are included.

## Slide 4
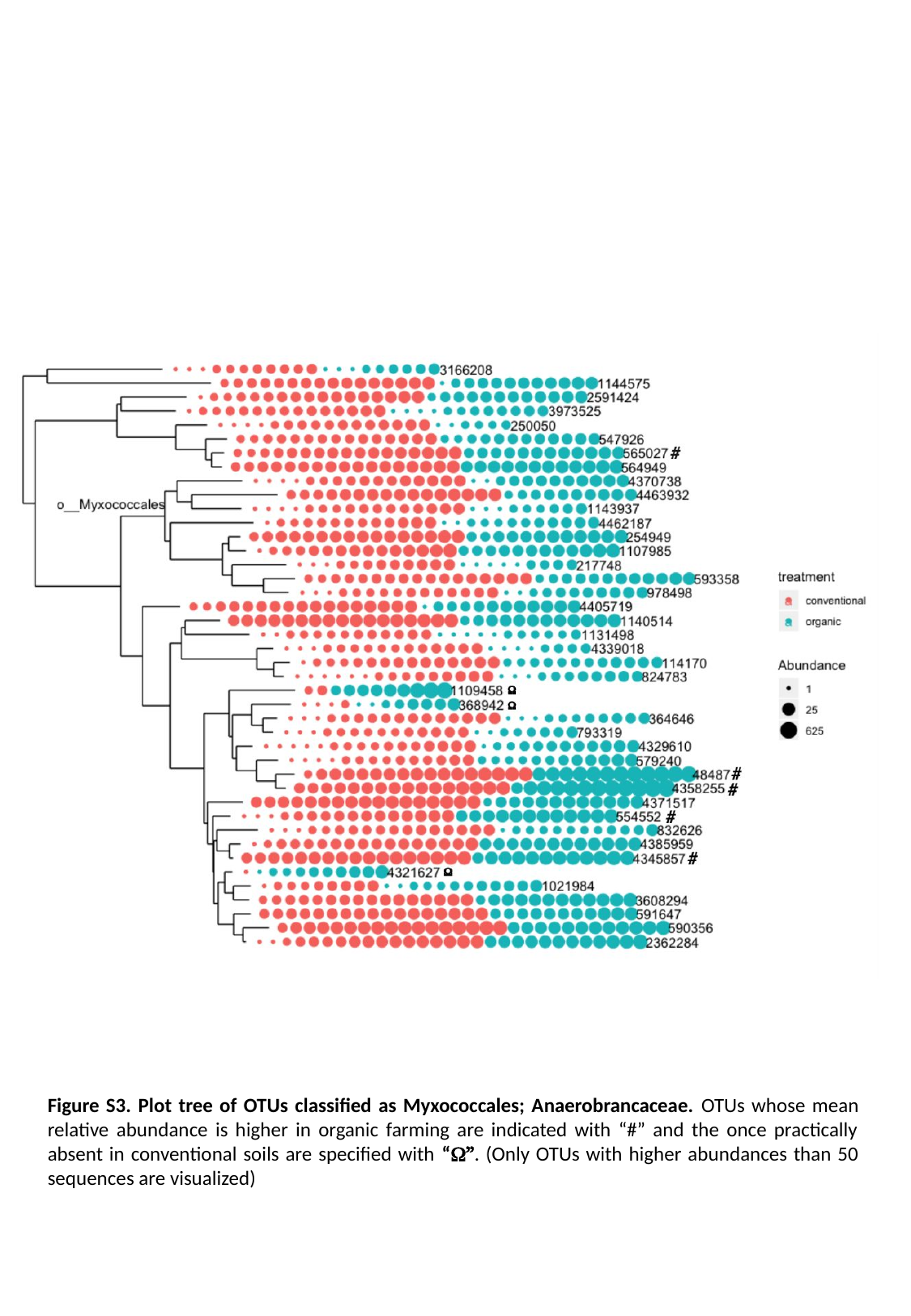

Figure S3. Plot tree of OTUs classified as Myxococcales; Anaerobrancaceae. OTUs whose mean relative abundance is higher in organic farming are indicated with “#” and the once practically absent in conventional soils are specified with “W”. (Only OTUs with higher abundances than 50 sequences are visualized)

## Slide 5
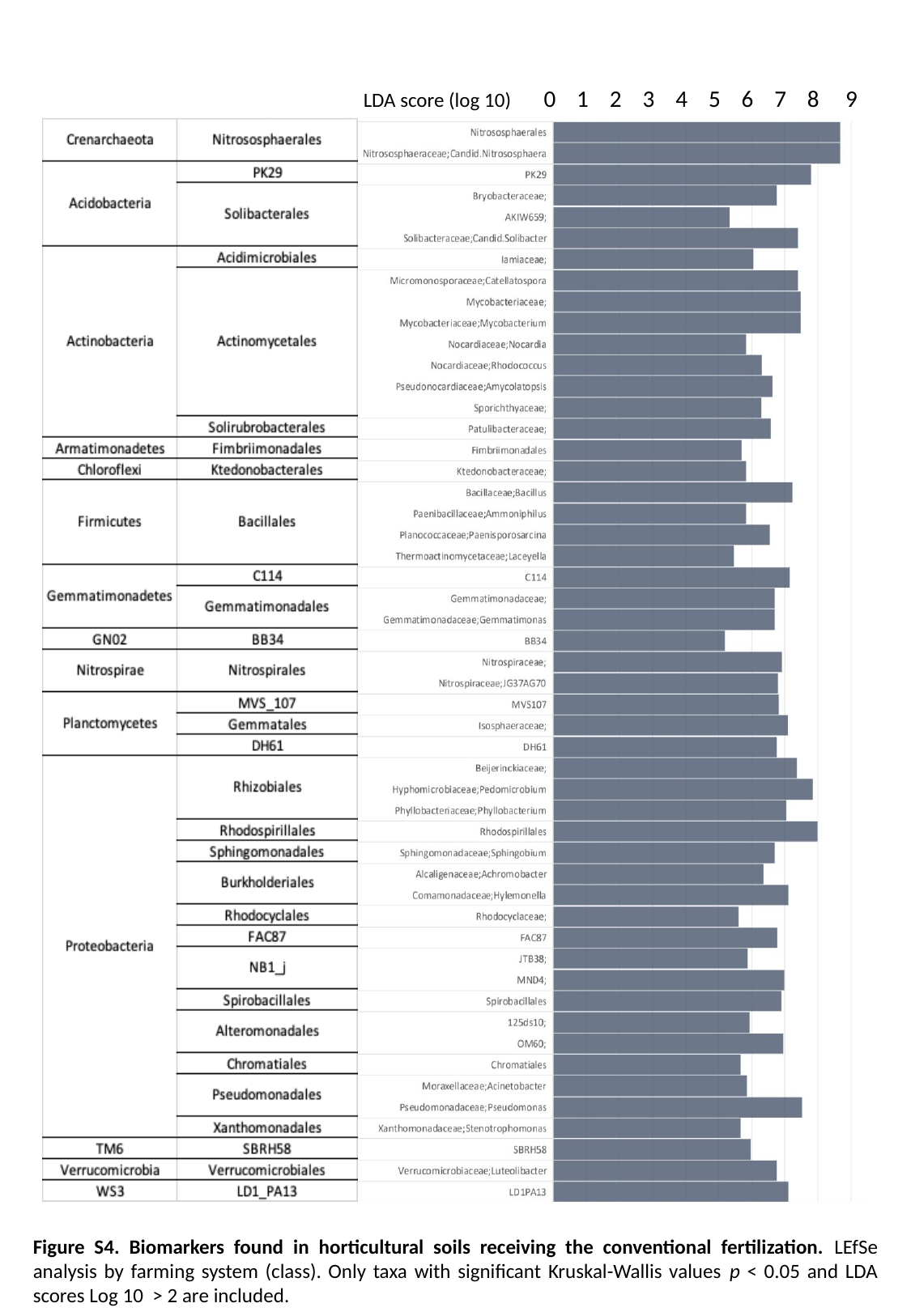

LDA score (log 10) 0 1 2 3 4 5 6 7 8 9
Figure S4. Biomarkers found in horticultural soils receiving the conventional fertilization. LEfSe analysis by farming system (class). Only taxa with significant Kruskal-Wallis values p < 0.05 and LDA scores Log 10 > 2 are included.

## Slide 6
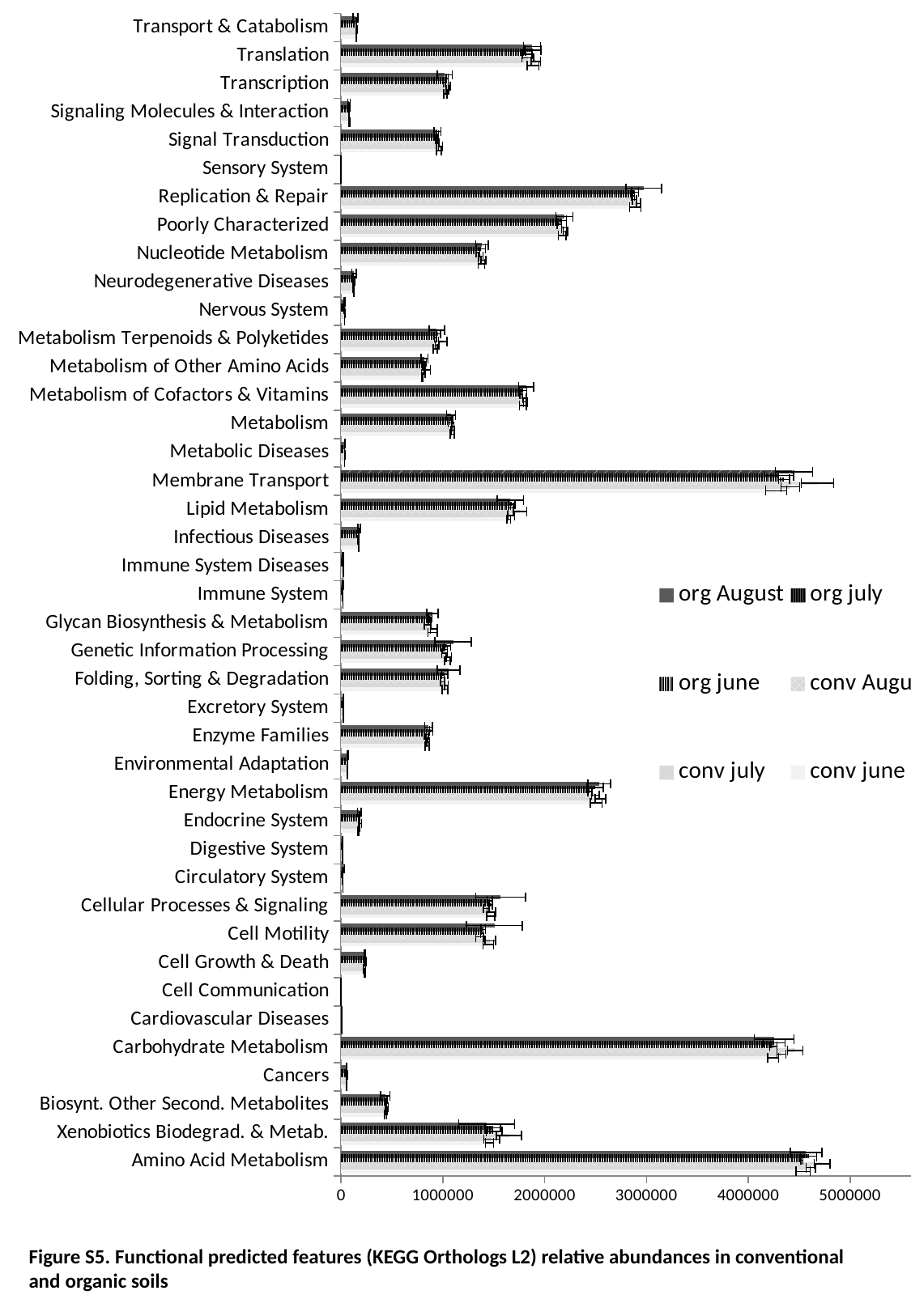

### Chart
| Category | conv june | conv july | conv August | org june | org july | org August |
|---|---|---|---|---|---|---|
| Amino Acid Metabolism | 4538287.33333333 | 4615643.16666667 | 4726116.2 | 4526093.33333333 | 4592187.66666667 | 4568055.16666667 |
| Xenobiotics Biodegrad. & Metab. | 1458481.16666667 | 1478631.5 | 1650237.8 | 1504253.0 | 1491914.66666667 | 1429674.0 |
| Biosynt. Other Second. Metabolites | 437861.6666666667 | 445530.8333333333 | 454841.8 | 439834.3333333333 | 448501.0 | 433577.3333333333 |
| Cancers | 54409.0 | 54146.66666666662 | 56047.6 | 60310.0 | 54164.0 | 55672.83333333334 |
| Carbohydrate Metabolism | 4242840.5 | 4325947.0 | 4460854.4 | 4245446.66666667 | 4254003.66666667 | 4253505.5 |
| Cardiovascular Diseases | 6449.833333333329 | 6147.833333333329 | 6827.2 | 8090.0 | 6718.66666666667 | 5735.5 |
| Cell Communication | 472.5 | 392.8333333333333 | 419.6 | 275.0 | 517.0 | 416.6666666666666 |
| Cell Growth & Death | 232124.5 | 232560.3333333333 | 233116.2 | 241212.0 | 233123.3333333333 | 233440.0 |
| Cell Motility | 1445740.0 | 1466027.16666667 | 1361385.2 | 1383726.0 | 1396822.33333333 | 1506898.16666667 |
| Cellular Processes & Signaling | 1470159.16666667 | 1475781.83333333 | 1427184.2 | 1468707.33333333 | 1460497.66666667 | 1567685.16666667 |
| Circulatory System | 17511.5 | 17449.0 | 15757.6 | 17837.66666666667 | 16728.0 | 20452.33333333331 |
| Digestive System | 15817.66666666667 | 15491.33333333333 | 14902.2 | 16513.33333333331 | 15832.33333333333 | 15493.83333333333 |
| Endocrine System | 172187.6666666667 | 177340.3333333333 | 191181.6 | 174241.3333333333 | 181044.6666666667 | 179490.1666666667 |
| Energy Metabolism | 2506006.83333333 | 2546622.33333333 | 2498942.6 | 2444596.33333333 | 2493300.0 | 2534762.33333333 |
| Environmental Adaptation | 62658.5 | 64165.33333333334 | 62405.0 | 62475.33333333334 | 63237.66666666662 | 65378.0 |
| Enzyme Families | 846311.5 | 857261.166666667 | 845663.0 | 840336.666666667 | 884845.3333333334 | 861138.166666667 |
| Excretory System | 20080.0 | 20861.0 | 21403.6 | 19262.66666666667 | 20291.33333333331 | 19873.0 |
| Folding, Sorting & Degradation | 1020910.0 | 1034614.5 | 999269.4 | 995914.0 | 1017623.33333333 | 1057190.33333333 |
| Genetic Information Processing | 1046017.66666667 | 1059177.83333333 | 1017044.0 | 1022416.66666667 | 1043768.66666667 | 1101188.16666667 |
| Glycan Biosynthesis & Metabolism | 900187.666666667 | 910418.8333333334 | 850942.6 | 870046.0 | 876192.3333333334 | 898045.3333333334 |
| Immune System | 17395.83333333331 | 17563.0 | 16695.2 | 18055.0 | 18175.33333333331 | 19717.5 |
| Immune System Diseases | 19850.5 | 20455.16666666667 | 22289.4 | 20319.0 | 21414.66666666667 | 19169.83333333331 |
| Infectious Diseases | 173413.0 | 174053.5 | 165986.8 | 175099.3333333333 | 174751.3333333333 | 176471.1666666667 |
| Lipid Metabolism | 1647298.83333333 | 1671740.5 | 1758903.0 | 1668767.66666667 | 1676983.33333333 | 1664600.33333333 |
| Membrane Transport | 4273400.0 | 4413938.66666667 | 4679660.0 | 4352824.33333333 | 4300481.0 | 4450782.66666667 |
| Metabolic Diseases | 34009.83333333334 | 34514.66666666662 | 35546.8 | 33534.66666666662 | 34307.66666666662 | 34545.33333333334 |
| Metabolism | 1090817.33333333 | 1098986.33333333 | 1098411.8 | 1072237.33333333 | 1091914.33333333 | 1079878.16666667 |
| Metabolism of Cofactors & Vitamins | 1785623.5 | 1812198.83333333 | 1803001.4 | 1765789.33333333 | 1787773.66666667 | 1818736.5 |
| Metabolism of Other Amino Acids | 797811.3333333334 | 808113.166666667 | 847623.4 | 814961.0 | 818469.3333333334 | 820312.8333333334 |
| Metabolism Terpenoids & Polyketides | 926577.5 | 943260.0 | 999458.0 | 926509.3333333334 | 955959.3333333334 | 940867.0 |
| Nervous System | 33237.0 | 34458.5 | 37418.4 | 32914.0 | 33389.66666666662 | 33037.16666666662 |
| Neurodegenerative Diseases | 124117.6666666667 | 122818.6666666667 | 118661.0 | 130853.0 | 121387.6666666667 | 128848.3333333333 |
| Nucleotide Metabolism | 1379209.16666667 | 1397948.5 | 1372250.6 | 1345897.33333333 | 1374489.33333333 | 1386002.83333333 |
| Poorly Characterized | 2171042.0 | 2203744.0 | 2190421.6 | 2144884.66666667 | 2165272.33333333 | 2194448.0 |
| Replication & Repair | 2888784.83333333 | 2921136.0 | 2882385.8 | 2869074.66666667 | 2888260.0 | 2973117.0 |
| Sensory System | 153.8333333333333 | 130.1666666666667 | 138.2 | 90.33333333333327 | 170.6666666666667 | 137.1666666666667 |
| Signal Transduction | 961156.8333333334 | 977619.0 | 949926.8 | 938539.3333333334 | 947341.0 | 945764.666666667 |
| Signaling Molecules & Interaction | 79946.66666666667 | 80828.33333333333 | 81596.8 | 80972.0 | 81601.0 | 76957.16666666667 |
| Transcription | 1024639.83333333 | 1045548.5 | 1062405.8 | 1013981.66666667 | 1042628.66666667 | 1020200.0 |
| Translation | 1887194.5 | 1914967.83333333 | 1834396.4 | 1827930.66666667 | 1883706.0 | 1875778.16666667 |
| Transport & Catabolism | 146856.5 | 148983.8333333333 | 157758.0 | 147843.6666666667 | 150971.6666666667 | 143517.6666666667 |Figure S5. Functional predicted features (KEGG Orthologs L2) relative abundances in conventional and organic soils

## Slide 7
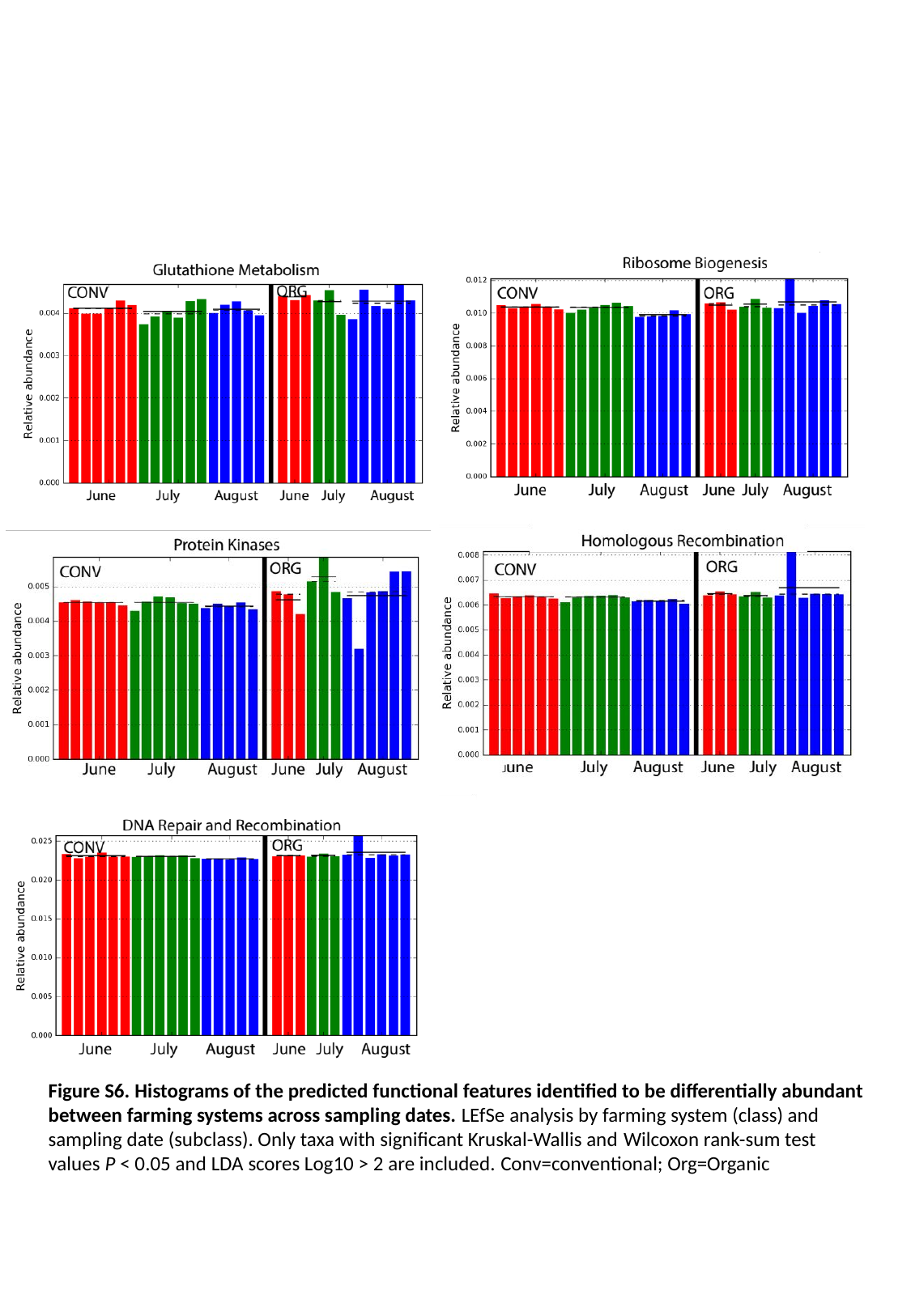

Figure S6. Histograms of the predicted functional features identified to be differentially abundant between farming systems across sampling dates. LEfSe analysis by farming system (class) and sampling date (subclass). Only taxa with significant Kruskal-Wallis and Wilcoxon rank-sum test values P < 0.05 and LDA scores Log10 > 2 are included. Conv=conventional; Org=Organic
